# Combined Use of Bicyclol and Berberine Alleviates Mouse Nonalcoholic Fatty Liver Disease

**DOI:** 10.3389/fphar.2022.843872

**Published:** 2022-02-16

**Authors:** Hu Li, Nan-Nan Liu, Jian-Rui Li, Biao Dong, Mei-Xi Wang, Jia-Li Tan, Xue-Kai Wang, Jing Jiang, Lei Lei, Hong-Ying Li, Han Sun, Jian-Dong Jiang, Zong-Gen Peng

**Affiliations:** ^1^ Institute of Medicinal Biotechnology, Chinese Academy of Medical Sciences and Peking Union Medical College, Beijing, China; ^2^ Key Laboratory of Biotechnology of Antibiotics, The National Health and Family Planning Commission (NHFPC), Institute of Medicinal Biotechnology, Chinese Academy of Medical Sciences and Peking Union Medical College, Beijing, China

**Keywords:** nonalcoholic fatty liver disease, bicyclol, berberine, combination, lipid metabolism, gut microbiota

## Abstract

Nonalcoholic fatty liver disease (NAFLD), ranging from simple steatosis to nonalcoholic steatohepatitis (NASH), is a liver disease worldwide without approved therapeutic drugs. Anti-inflammatory and hepatoprotective drug bicyclol and multi-pharmacological active drug berberine, respectively, have shown beneficial effects on NAFLD in murine nutritional models and patients, though the therapeutic mechanisms remain to be illustrated. Here, we investigated the combined effects of bicyclol and berberine on mouse steatosis induced by Western diet (WD), and NASH induced by WD/CCl_4_. The combined use of these was rather safe and better reduced the levels of transaminase in serum and triglycerides and cholesterol in the liver than their respective monotherapy, accompanied with more significantly attenuating hepatic inflammation, steatosis, and ballooning in mice with steatosis and NASH. The combined therapy also significantly inhibited fibrogenesis, characterized by the decreased hepatic collagen deposition and fibrotic surface. As per mechanism, bicyclol enhanced lipolysis and β-oxidation through restoring the p62-Nrf2-CES2 signaling axis and p62-Nrf2-PPARα signaling axis, respectively, while berberine suppressed *de novo* lipogenesis through downregulating the expression of acetyl-CoA carboxylase and fatty acid synthetase, along with enrichment of lipid metabolism-related Bacteroidaceae (family) *and Bacteroides* (genus). Of note, the combined use of bicyclol and berberine did not influence each other but enhanced the overall therapeutic role in the amelioration of NAFLD. *Conclusion*: Combined use of bicyclol and berberine might be a new available strategy to treat NAFLD.

## Introduction

Nonalcoholic fatty liver disease (NAFLD), a common liver disease affecting a quarter of the world’s population, includes nonalcoholic fatty liver (NAFL) characterized by mere excessive lipid accumulation in the liver and nonalcoholic steatohepatitis (NASH), characterized by inflammation and/or fibrosis and even cirrhosis (Eslam et al., 2020; Huang et al., 2021). NAFLD is recently referred to as metabolic-associated fatty liver disease (MAFLD), which also includes extrahepatic complications, such as obesity, type 2 diabetes, and cardiovascular and cardiac diseases (Eslam et al., 2020; Liu et al., 2020). Abnormality of hepatic lipid metabolism, including lipogenesis, lipolysis, and fatty acid β-oxidation, links with the occurrence of progressive NAFLD. Lipogenesis is a normal synthesis of triglycerides (TG) and fatty acids from acetyl coenzyme A (acetyl-CoA). During the *de novo* lipogenesis, acetyl-CoA is carboxylated to malonyl-CoA by the rate-limiting enzyme acetyl-CoA carboxylase (ACC) and subsequently converted by a multi-step reaction into long-chain fatty acid by fatty acid synthase (FAS) (Mashima et al., 2009; Gathercole et al., 2011), while excessive synthesis of TG is one of the main causative factors for NAFLD (Alves-Bezerra and Cohen, 2017). Conversely, lipolysis is a normal breakdown process of TG to form fatty acids and glycerol and abnormal lipolysis is another mechanism for NAFLD. Apart from classical proteins related with lipolysis, such as adipose triglyceride lipase (ATGL) and hormone-sensitive lipase (HSL), carboxylesterase 2 (CES2) is recently discovered as an efficient diglyceride, monoglyceride, and triglyceride hydrolase, which plays a causal role in the development of obesity and fatty liver diseases in human and murine (Li et al., 2016; Chalhoub et al., 2021). Hepatic mitochondrial β-oxidation is a major pathway for the catabolism of fatty acids, which is mainly regulated by peroxisome proliferator-activated receptor α (PPARα) and subsequent regulated expression of targeted genes (Wan et al., 2020). Consequently, the abnormal hepatic lipid metabolism is involved in sustained inflammation response and liver injury, further leading to the progression of NAFLD (Dong et al., 2021; Orabi et al., 2021). Apart from the aberrant hepatic lipid metabolism, gut microbiome-mediated alteration of immunity, inflammation, and metabolism are also involved in the regulation of NAFLD (He et al., 2021).

Currently, no specific drug has been approved to treat NAFLD, though several candidate drugs with different mechanisms of action have been tested in clinical trials (Brown et al., 2021), which target different pathophysiological pathways towards NAFLD, such as metabolic targets, inflammatory pathways, liver-gut axis, and antifibrotic targets. Because NAFLD is a multisystem disease with intricate pathological mechanisms, the therapeutic effect with a single agent is generally unsatisfactory (Huang et al., 2020; Brown et al., 2021; Zhu et al., 2021). Therefore, the combination of drugs with different mechanisms might provide potential opportunities to enhance the overall efficacy, and several clinical trials have been carried out to validate this conception (Dufour et al., 2020; Kessoku et al., 2020). Natural products were widely reported for the therapy of liver diseases (Li et al., 2019). Bicyclol, a synthetic compound that originated from the Chinese traditional herb *Schisandra chinensis*, is an approved drug for alleviating liver injuries accompanied by elevated transaminases caused by various etiologies, such as viruses, drugs, alcohol, chemicals, and immunogens (Liu, 2009; Li et al., 2018a; Li et al., 2018b). A few studies reported the therapeutic efficacy of bicyclol for NAFLD in clinic (Li et al., 2020) and in animal models (Zhao et al., 2021), though more data are needed to show its clinical benefits and the detailed mechanisms. Berberine, an approved antibacterial agent derived from a wide variety of Chinese traditional herbs, such as *Coptis chinensis* and *Berberis vulgaris* (Kong et al., 2020), has beneficial effects on NAFLD through diverse mechanisms, including the increase of insulin sensitivity, regulation of adenosine monophosphate-activated protein kinase (AMPK) pathway, improvement of mitochondrial function, and regulation of the gut microenvironment (Zhu et al., 2016; Wang et al., 2020). Therefore, considering current mono-therapeutic evidence of bicyclol and berberine and their distinguished mechanisms against NAFLD, we proposed a new potential strategy of their combined use to treat NAFLD. In this study, we demonstrated that the combination of bicyclol and berberine exerted better preventive and therapeutic effects than monotherapy for NAFLD in mice induced by Western diet (WD) or WD/CCl_4_. The detailed mechanisms are involved in the enhancement of lipolysis and β-oxidation by bicyclol, and suppression of lipogenesis by berberine, along with its regulation of the gut microbiome. Our study provided a new strategy for the treatment of NAFLD with the combined use of bicyclol and berberine.

## Materials and Methods

### Chemicals and Reagents

Bicyclol was from Beijing Union Pharmaceutical Company (Beijing, China) with a purity of over 99%. Berberine was purchased from InnoChem (Beijing, China) with a purity of over 97%. The purified low-fat, low-cholesterol diets (LFLC, LAD0011), Western diet (WD, TP26300122, contains 21.1% fat, 41% sucrose, 1.25% cholesterol), and WD mingled with low or high doses of bicyclol and berberine, and their reciprocal combination was produced by Trophic Animal Feed High-Tech Co., Ltd, China. D-fructose (Sigma, F0127), D-glucose (Sigma, G8270), and CCl_4_ (Tianjin Fuchen Chemical Reagent Factory) were used in the experiments. Assay kit for alanine aminotransferase (ALT, C009-2), aspartate transaminase (AST, C010-2), triglyceride (TG, A110-1), and cholesterol (CHO, A111-1) were from Nanjing Jiancheng Biotechnology Co., Ltd, China. Oleic acid (OA, O1008); palmitic acid (PA, P0500), bovine serum albumin (BSA, B2064), and Nile Red (N3013) were from Sigma-Aldrich. Methyl thiazolyl tetrazolium (MTT, M8180), phosphate buffer solution (PBS, P1020), and trypsin (G2161) were from Beijing Solarbio Science & Technology Co., Ltd.

### Animal Experiments

Male C57BL/6J mice (25–27 g) were from SPF (Beijing) Biotechnology Co., Ltd. and housed in a 12-h light/dark standard light cycle with free access to water and food. After 7 days of acclimation, mice were allocated for the preventive and therapeutic experiments. For the preventive experiment, mice were randomly divided into 10 groups, with 5 mice in each group. Mice in the drug-treated experimental group were fed with WD or WD mingled with low or high doses of bicyclol, berberine, and their reciprocal combination for 16 weeks. The low and high doses of bicyclol and/or berberine in diet were equivalently 50 and 200 mg/kg/day by gavage in mice. In parallel, high sugar drinking water with 23.1 g/L D-fructose and 18.9 g/L D-glucose were fed to the experimental group while mice in control were provided with an LFLC diet and regular drinking water. Food intake and body weight were recorded every week. In the therapeutic experiment, WD/CCl_4_ (WD plus intraperitoneally injection of CCl_4_ at the dose of 0.2 ml/kg in corn oil once per week) was used to induce mouse NASH. After 4 weeks of induction by WD/CCl_4_, mice were divided into 10 groups (five to nine mice in each group) and fed with the corresponding diet and water as conducted in the preventive experiment for another 8 weeks. At the end of the experiments, animals were fasted for 6 h, and then blood samples were collected. For liver biochemistry and subsequent mechanism study, intralobular pieces of liver were quickly frozen in liquid nitrogen and then stored at −80°C. For histological analyses, liver slices were fixed with 4% paraformaldehyde (Servicebio, #G1101).

### Biochemical Analysis

The blood samples were centrifuged at 2,500 g for 10 min, and the serum was collected. Serum levels of ALT and AST were detected using commercial assay kits. For analyses of liver biochemistry, mouse liver was homogenized, and the levels of hepatic TG and CHO were measured using assay kits according to the manufacturer’s instructions.

### Histological Analysis

The fixed liver tissues were conducted using hematoxylin and eosin (H&E) staining for evaluating liver inflammation, steatosis, and ballooning. The NAFLD classifications were assessed by two experts blindly according to the NAFLD activity score (NAS) criteria, which is a composite semi-quantitative score for steatosis (0–3), lobular inflammation (0–2), hepatocellular ballooning (0–2), and fibrosis (0–4) (Kleiner et al., 2005). The presence of steatosis was further confirmed with Oil Red O (ORO) staining in frozen sections using a standard protocol. Fibrosis was qualifiedly assessed using Masson’s Trichrome staining in paraffin-embedded sections, and ImageJ software was used to quantify the percentage of fibrosis surface in six fields of Masson’s Trichrome staining sections of each mouse.

### Cell Treatment and Cytotoxicity Assay

HepG2 cells were maintained in 5% CO_2_ at 37°C and cultured in Minimum Essential Medium (Gibico, C11095500) with 10% fetal bovine serum, 100 IU/ml penicillin and 100 mg/ml streptomycin (Gibico, 15140122). HepG2 cells were plated in a 96-well plate at the concentration of 3 × 10^4^/cm^2^. The cell viability was detected with an MTT assay after 0.1 mM free fatty acid (FFA, OA:PA = 2:1) and drug treatment for 24 h, and the data were calculated as described before (Zou et al., 2021).

### Nile Red Staining Assay

The cell-specific climbing slides were placed in 24-well plates and coated with rat tail collagen for 15 min. After being washed with PBS, 1 × 10^5^ cells/well HepG2 were seeded and incubated at 37°C and 5% CO_2_ for 24 h. The cells were induced with 0.1 mM FFA and simultaneously treated with 2 μM berberine, 2 μM bicyclol, or their combinations. After 24 h of treatment, the cells were washed with PBS and fixed with 3% paraformaldehyde for 10 min, followed by washing with PBS. Next, 0.5% Triton X-100 (Beyotime Biotechnology, ST795) was added for 15 min for membrane permeabilization and then washed with PBS twice. Nile Red solution at 10 μM concentration was incubated for 10 min. After being washed with PBS, the slides were sealed by mounting medium supplied with DAPI staining solution and dried in the dark. The images were acquired using Zeiss Axio observer X-cite series 120 microscope (Zeiss, Jena, Germany) at the magnification of ×63. The quantification of lipid droplets was performed using Image-Pro Plus 6.0.

### Western Blot

Liver homogenates or cell lysates were prepared in a protein extraction reagent (Thermo Scientific, 78510) with protease and phosphatase inhibitor cocktail (Targetmol, C0001 and C0004). The protein concentration was determined using BCA protein assay kit (Thermo Scientific, #23225) according to the manufacturer’s manual. Western blot was performed as previously described (Cheng et al., 2016; Huang et al., 2019). Briefly, 20–80 μg of proteins were separated by 10% SDS-PAGE and transferred to PVDF membranes. Membranes were blocked with 5% fat-free milk and incubated overnight at 4°C with antibodies against β-actin (CST, 3700S), CES2 (Abcam, ab215042), CES2 (Sangon Biotech, D263440), PPARα (HUABIO, EM1707-71), PPARα (Santa Cruz, sc-398394), Nrf2 (HUABIO, ER1706-41), p62 (HUABIO, EM0704), ACC (HUABIO, ET1609-77), FAS (HUABIO, R1706-8), ATGL (HUABIO, RT1058), HSL (CST, #4107), ACSL5 (HUABIO, ER60809), ACSL1 (HUABIO, ER60807), CD36 (Abcam, ab133625), and FABP1 (HUABIO, EM170403). The PVDF band was then incubated with the corresponding HRP-conjugated secondary antibody, and the signal of the target proteins was detected with ECL chemiluminescence detection kit (Vazyme, #E412) using the ChemiDoc MP imaging system (Bio-Rad). Signal intensity was scanned with Gel-Pro analyzer, and a ratio of the protein of interest to the internal control protein Actin was calculated and normalized as 1.00 for the control group.

### Intestinal Microbiological Analysis

Five representative groups in the therapeutic experiment were selected to perform gut microbiological analysis, and a total of 25 mice fecal samples with five in each group were collected and subsequently analyzed using 16S rRNA gene sequencing by Oebiotech (Shanghai, China). Briefly, genomic DNA was isolated from samples using the DNeasy PowerSoil Kit (QIAGEN) and amplified using specific sequences of primers for the V3-V4 region of 16S rRNA (343F-5ʹ TACGGRAGGCAGCAG 3ʹ and 798R-5ʹ AGGGTATCTAATCCT 3ʹ). The amplified products were checked by 1% agarose gel electrophoresis and purified using AMPure XP beads (Agencourt), and then amplified in another round of PCR as described above. The final amplicon was quantified using the Qubit quantification system (Life Technologies) and purified again. Equal amounts of purified amplicon were pooled for subsequent sequencing using the Illumina MiSeq System (Illumina Inc., San Diego, CA, USA). For bioinformatics analysis, the obtained raw FASTQ sequencing files were preprocessed using the QIIME software (version 1.8.0). Clean reads were subjected to primer sequence removal and clustering to generate operational taxonomic units (OTUs) using the Vsearch software (version 2.4.2) with 97% similarity cutoff. Based on the rarefied OTU counts, the community structure, the alpha diversity (presented as Chao index), and beta diversity [presented as unweighted UniFrac distances and principal coordinate analysis (PCoA)] were analyzed to plot the similarity or difference in the composition of the sample community. Functional inference associated with lipid metabolism was identified using Kyoto Encyclopedia of Gene and Genomes (KEGG) pathways.

### Statistical Analyses

The data were presented as mean ± standard deviation (SD) and representative figures. Statistical analysis was performed using SPSS17.0 or GraphPad Prism 8 and analyzed by Student’s *t*-test or analysis of variance (ANOVA) followed by Student–Newman–Keuls (SNK) post hoc tests. Kruskal–Wallis H test and Mann-Whitney *U* test were used for the nonparametric test. The value of statistical significance was set as *p < 0.05 or **p < 0.01.

## Results

### Combination of Bicyclol and Berberine Exerts Better Preventive Effects in Mice With NAFL Induced by Western Diet

**FIGURE 1:**
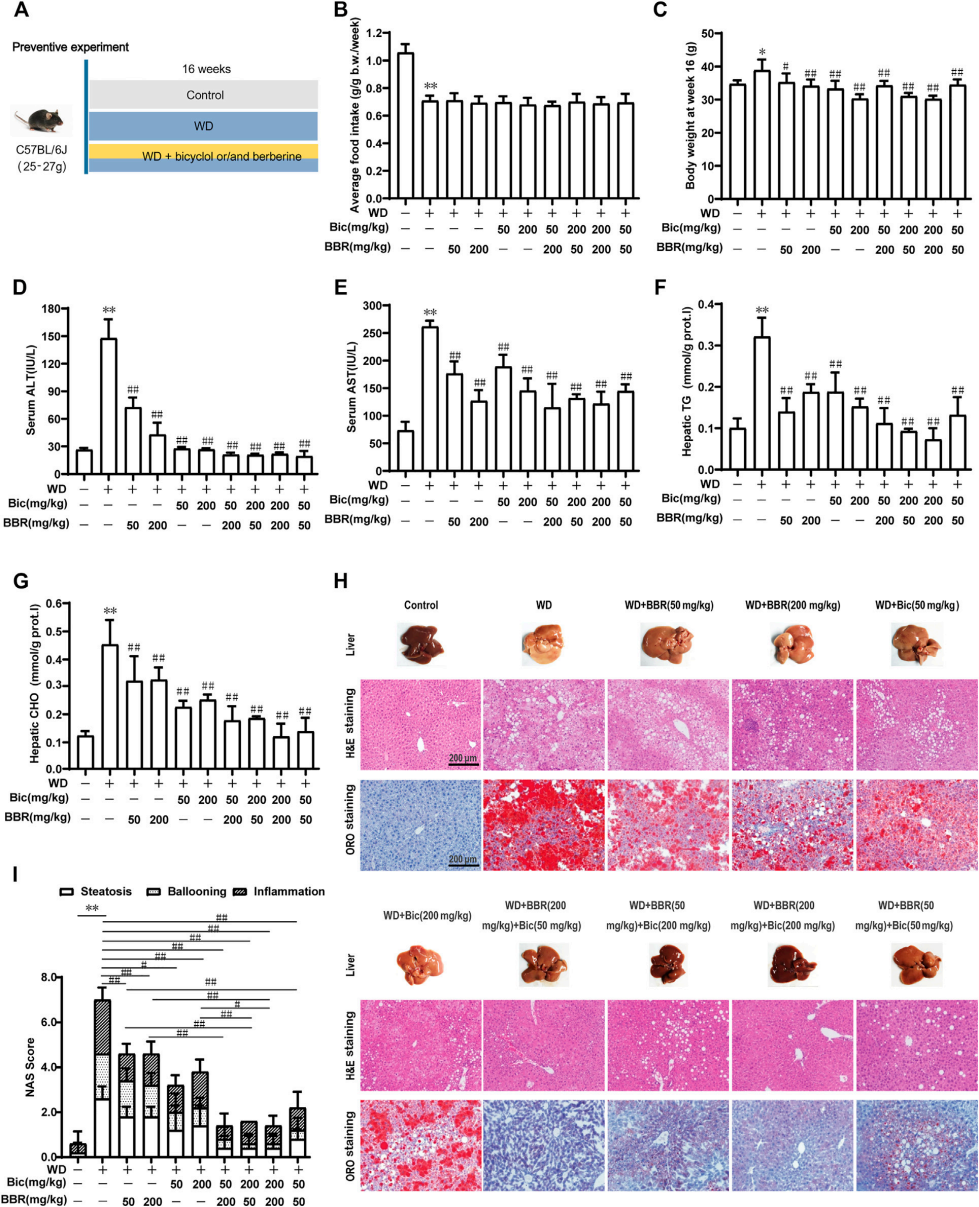
Combination of bicyclol (Bic) and berberine (BBR) exerts better preventive effects in mice with NAFL induced by Western diet. Male C57BL/6J mice were treated by free feeding with Western diet (WD) or WD mingled with bicyclol or/and berberine **(A)**. The average food intake during the whole experiment **(B)** and body weight at week 16 **(C)** were recorded. At week 16, ALT **(D)** and AST **(E)** in serum were detected, and TG **(F)** and CHO **(G)** in the liver were measured. Liver histopathology was evaluated using H&E and ORO staining **(H)** and quantified with NAS score criteria **(I)**. Results were presented as mean ± SD. n = 5 for each group, ^*^p < 0.05 and ^**^p < 0.01, WD-induced model group vs. the control group; ^#^p < 0.05 and ^##^p < 0.01 vs. the WD-induced model group or monotherapy group.

To analyze the combined therapeutic effect of bicyclol and berberine, we first evaluated their preventive efficacy in a murine nutritional model of NAFL. Briefly, C57BL/6J mice were treated with WD or WD mingled with low/high dose of bicyclol and/or berberine by free feeding for 16 weeks, and the low-fat, low-cholesterol (LFLC) diet was set as normal diet control (Figure 1A). There were no toxic phenotypes (data not shown) and no significant difference in average food intake during the whole experiment among the WD-induced group and drug-treated groups, though the mice treated with WD decreased the food intake when compared with normal diet control (Figure 1B). Treatment with WD slightly increased the body weight of mice, while it was decreased after monotherapy and combination administration (Figure 1C). ALT and AST in serum and TG and CHO in the liver were notably ascended after WD induction (Figures 1D–G), suggesting that hepatic lipid accumulation induced by excessive nutrition was associated with impaired liver function. In contrast, all the biochemical indicators were decreased after bicyclol and berberine treatment, especially their combined treatment (Figures 1D–G). These results demonstrated that the combined use of bicyclol and berberine might possess better biochemical prevention of WD-induced liver injury and steatosis without significant toxicity.

Pathologically, treatment of WD induced a pale and rough appearance of the liver, while bicyclol or berberine mono-treatment ameliorated the liver appearance, and the combined use of these improved it more significantly at both low and high doses (Figure 1H, Liver). H&E and ORO staining of the livers treated with WD exhibited histopathological lesions with early liver disease, paralleled with significant hepatic inflammation, steatosis, and ballooning, while monotherapy with bicyclol or berberine significantly alleviated the histopathological lesions and the combined treatment exhibited better pharmacologic effect (Figure 1H, H&E and ORO staining). The effects were also confirmed by quantitation with the NAS score criterion (Figure 1I). Therefore, the combined use of bicyclol and berberine presented more superior preventive effects in a murine nutritional model of NAFL than individual treatment, which agrees with our putative strategy, though the dose-dependent efficacy of the two drugs was less than ideal.

### Combination of Bicyclol and Berberine Demonstrates Better Therapeutic Effects on NASH Induced by Western Diet Plus CCl_4_

**FIGURE 2:**
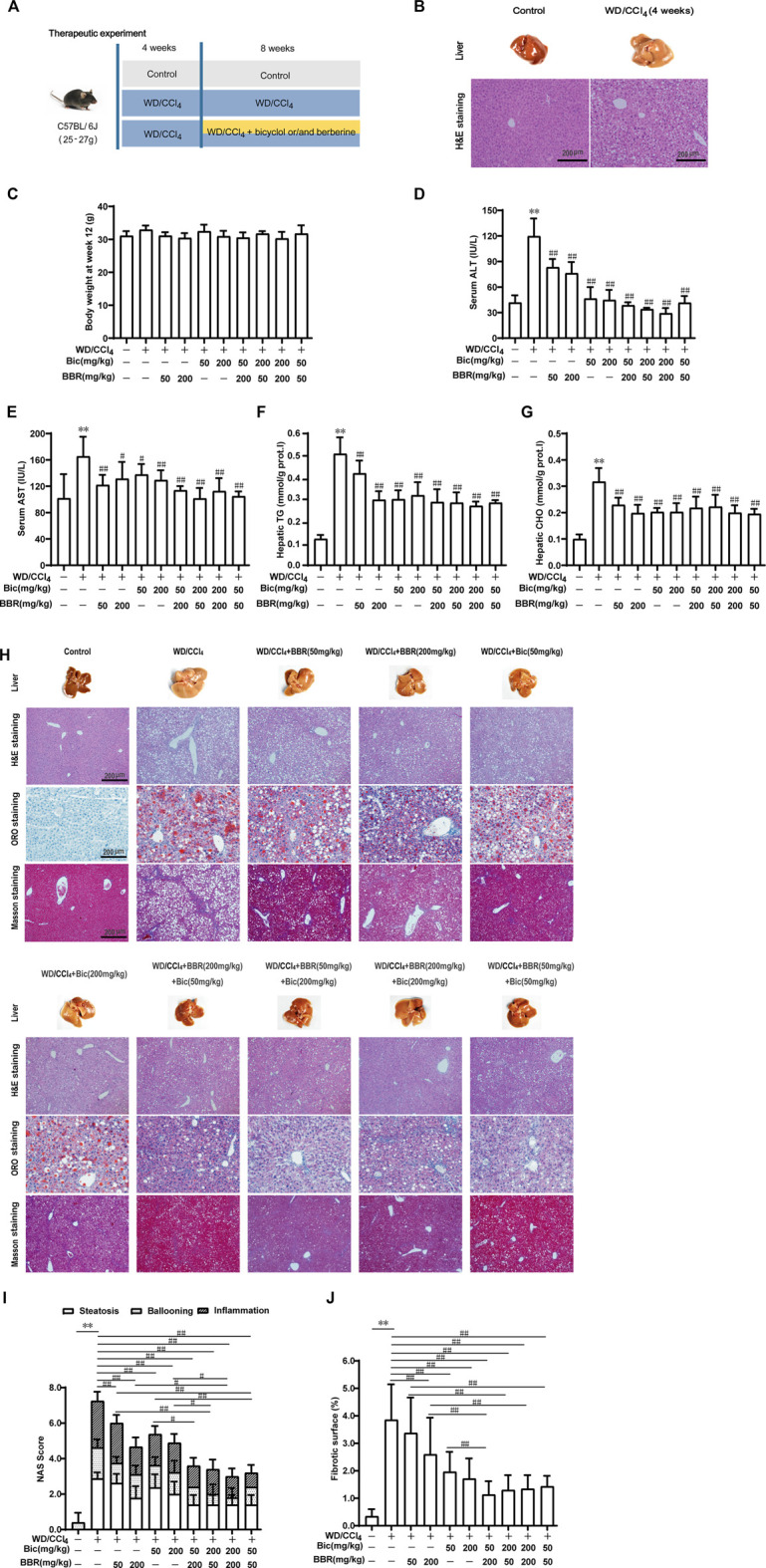
Combination of bicyclol and berberine demonstrates better therapeutic effects on NASH in mice induced by WD/CCl_4_. Male C57BL/6J mice were freely fed with Western diet plus intraperitoneal injection of 0.2 ml/kg CCl_4_ (WD/CCl_4_) once per week for 4 weeks **(A)**. Liver steatosis was performed with H&E staining at week 4 **(B)**. Then the mice were continuously treated with WD/CCl_4_ or WD/CCl_4_ plus drug for 8 weeks. The body weight **(C)** was recorded. ALT **(D)** and AST **(E)** in serum and TG **(F)** and CHO **(G)** in the liver were measured. Liver histopathology was evaluated using H&E, ORO, and Masson’s trichrome staining **(H)** and quantified with NAS score criteria **(I)** and fibrotic surface **(J)**. Results were presented as mean ± SD. n = 5–9 for each group, *p < 0.05 and **p < 0.01, WD/CCl_4_-induced model group vs. the control group; ^#^p < 0.05 and ^##^p < 0.01 vs. the WD/CCl_4_-induced model group or monotherapy group.

Given the preferable preventive effect against hepatic steatosis by the combination of bicyclol and berberine, we further investigated their therapeutic effects on steatohepatitis (Figure 2A). C57BL/6J mice were freely fed with WD and intraperitoneally injected with 0.2 ml/kg CCl_4_ once per week for 4 weeks to induce hepatic steatosis (Figure 2B). Then, the mice were continuously treated with WD/CCl_4_ or plus low/high dose of bicyclol or/and berberine for 8 weeks. LFLC diets plus corn oil injection were conducted as normal control. There was no significant difference in body weight among the groups at week 12 (Figure 2C). However, ALT and AST in serum, and TG and CHO in the liver were significantly elevated by the introduction of WD/CCl_4_ (Figures 2D–G). Bicyclol or berberine monotherapy showed efficacy to reduce these biochemical indicators, and the combined treatment of bicyclol and berberine reversed their elevations, with an overall tendency of more excellent potency than monotherapy (Figures 2D–G). Histopathological results further confirmed that mice were progressed to NASH induced by WD/CCl_4_, showing aberrant liver appearance, hepatic inflammation, steatosis, ballooning, and collagen deposition (Figure 2H). Bicyclol or berberine monotherapy showed a high beneficial effect to alleviate the pathological outcomes, and the combined therapy was more effective than monotherapy (Figure 2H), which was further verified by quantification of NAS score (Figure 2I) and fibrotic surface (Figure 2J). Therefore, based on the available results, combined therapy with bicyclol and berberine appears more effective than monotherapy in the treatment of NASH.

### Bicyclol With or Without Berberine Enhances Lipolysis and β-Oxidation Through p62-Nrf2-CES2/PPARα Signaling Axis

**FIGURE 3:**
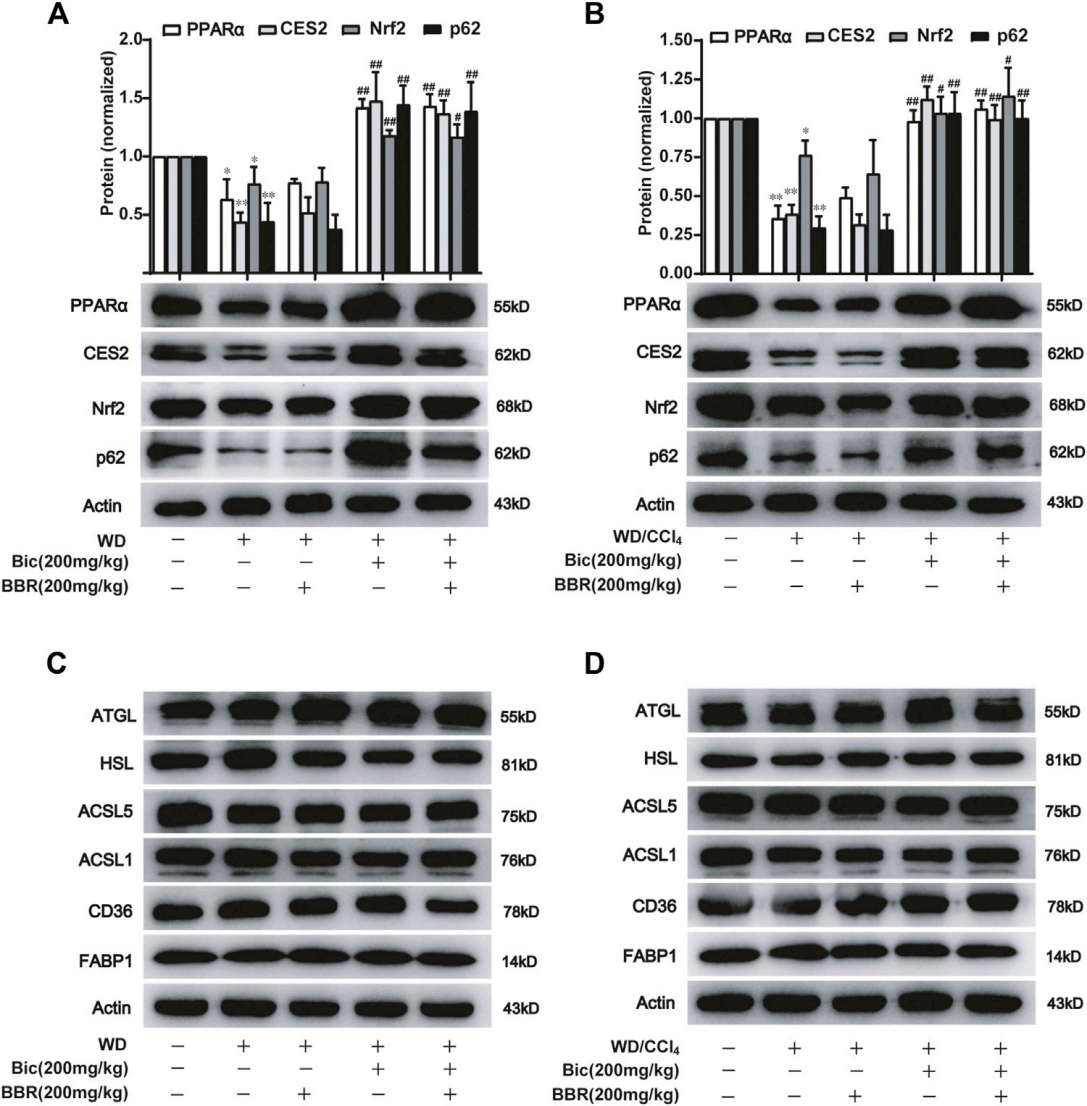
Bicyclol with or without berberine enhances the lipolysis and β-oxidation through p62-Nrf2-CES2/PPARα axis. Male C57BL/6J mice were freely fed with WD or WD plus bicyclol and/or berberine for 16 weeks **(A,C)**, or induced with WD/CCl_4_ for 4 weeks and then treated with drugs for 8 weeks **(B,D)**. The protein levels of PPARα, CES2, Nrf2, p62, CD36, FABP1, ATGL, HSL, ACSL1, and ACSL5 were detected with Western blot and quantified using a Gel-Pro analyzer. Results were presented as mean ± SD, and a representative band was shown. n = 5–9 for each group, *p < 0.05 and **p < 0.01, the model group vs. control group; ^#^p < 0.05 and ^##^p < 0.01 vs. the model group. PPARα, proliferator-activated receptor α; CES2, carboxylesterase 2; Nrf2, NF-E2-related factor 2; p62, ubiquitin-binding protein p62; CD36, cluster determinant 36; FABP1, fatty acid binding protein 1; ATGL, adipose triglyceride lipase; HSL, hormone-sensitive lipase; ACSL, acyl-CoA synthetase long chain family member.

Based on their respective therapeutic roles in clinic and the better improvement of NAFLD by combination of bicyclol and berberine in our animal experiments, we speculated that they might alleviate NAFLD through different mechanisms. Therefore, we first detected the expression of some potential lipid metabolism-related proteins in the high-dose and the combined groups which generally possessed the most significant pharmacologic effects (Figure 1, 2). Among these potential targets, PPARα, a key nuclear receptor that promotes β-oxidation, was markedly decreased in both WD-induced steatosis mice (Figure 3A) and WD/CCl_4_-induced NASH mice (Figure 3B), while bicyclol with or without berberine increased its expression. In contrast, compared with WD-induced steatosis and WD/CCl_4_-induced NASH model groups, berberine alone did not change the protein expression of PPARα, and it also did not disturb the effect of bicyclol (Figures 3A, B), suggesting that the enhancement of PPARα-mediated β-oxidation is exclusively the mechanism of bicyclol. Similarly, CES2 was downregulated in the two model groups, while bicyclol but not berberine reversed its expression (Figures 3A, B). As the promoting roles for lipolysis by CES2 has been well illustrated (Li et al., 2016; Chalhoub et al., 2021), we thus speculate that bicyclol could also enhance the lipolysis process. Of note, PPARα and CES2 were downstream targets of p62-Nrf2 signaling (Zhang et al., 2012; Baker et al., 2015; Tao et al., 2021). Our results further verified the protein expressions of p62 and Nrf2 were reduced significantly in WD- and WD/CCl_4_-induced model groups, while bicyclol but not berberine increased their expressions (Figures 3A, B). However, in WD- and WD/CCl_4_-induced mouse models, the levels of free fatty acid uptake-related proteins CD36 and FABP1, lipolysis-related proteins ATGL and HSL, and β-oxidation-related proteins ACSL1 and ACSL5 were not changed significantly and were not altered by the treatment of bicyclol, berberine, and their combination (Figures 3C, D). Therefore, these results showed that promoting for lipolysis through inhibiting the p62-Nrf2-CES2 signaling axis and enhancing for β-oxidation through increasing p62-Nrf2-PPARα signaling axis are the mechanisms of bicyclol but not berberine to alleviate NAFLD.

### Berberine With or Without Bicyclol Suppresses the *de Novo* Lipogenesis by Inhibiting Hepatic ACC and FAS Expressions

**FIGURE 4:**
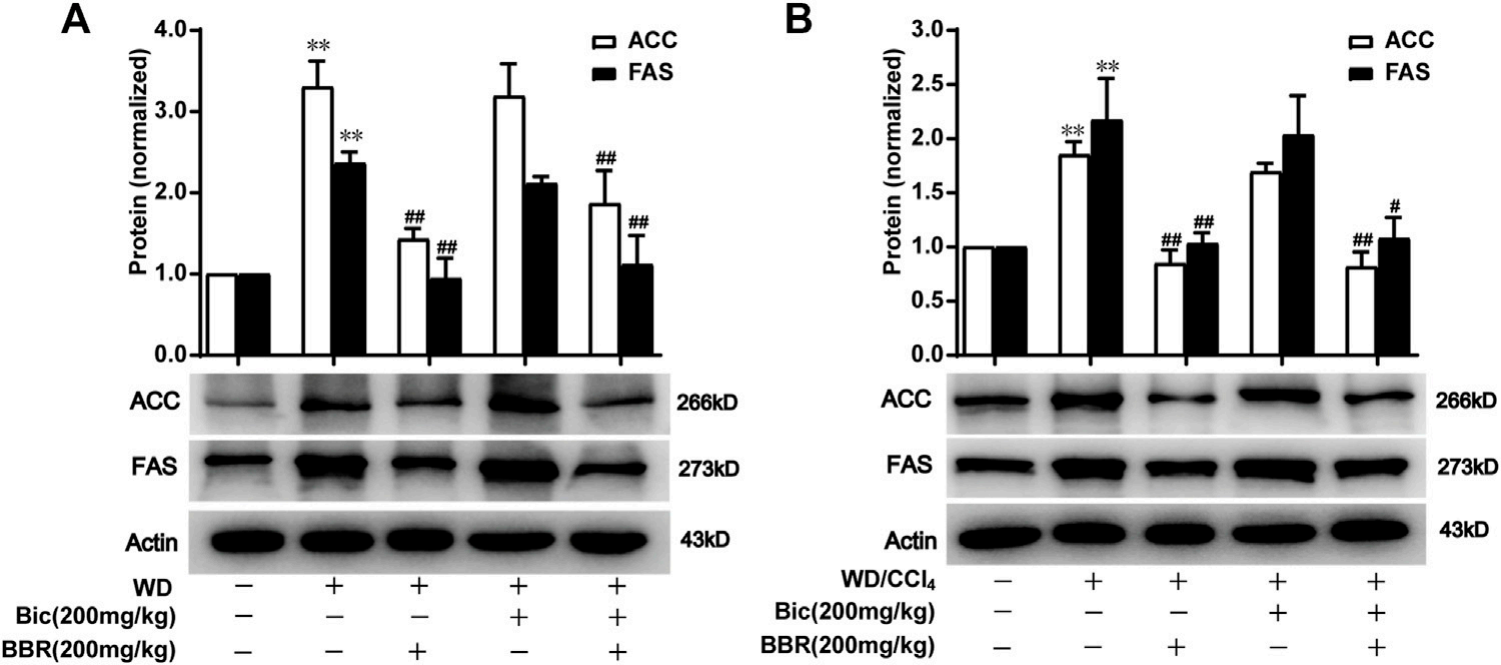
Berberine with or without bicyclol suppresses *de novo* lipogenesis by inhibiting the protein expression of ACC and FAS. Male C57BL/6J mice were freely fed with WD or WD plus bicyclol and/or berberine for 16 weeks **(A)**, or induced with WD/CCl_4_ for 4 weeks and then treated with drugs for 8 weeks **(B)**. The protein expressions of ACC and FAS were detected and quantified. Results were presented as mean ± SD, and a representative band was shown. n = 5–9 for each group, *p < 0.05 and **p < 0.01, the model group vs. control group; ^#^p < 0.05 and ^##^p < 0.01 vs. the model group. ACC, acetyl-CoA carboxylase; FAS, fatty acid synthase.

ACC catalyzes the fatty acid synthesis related carboxylation of acetyl-CoA to malonyl-CoA, and FAS catalyzes the synthesis of long-chain saturated fatty acids (Mashima et al., 2009; Gathercole et al., 2011), which are involved in *de novo* syntheses of TG and subsequent occurrence and progress of NAFLD (Alves-Bezerra and Cohen, 2017). Compared with the normal control group, ACC and FAS levels in the liver were increased after WD induction, while berberine but not bicyclol reversed the high expressions of ACC and FAS (Figure 4A), suggesting that WD promoted the *de novo* lipogenesis and berberine suppressed this process. Additionally, bicyclol did not disturb the inhibitory role of berberine on the expressions of ACC and FAS when combined with berberine (Figure 4A). Similar results were also demonstrated in the therapeutic experiment induced by WD/CCl_4_ (Figure 4B). However, berberine did not change other lipid metabolism-related protein expression, such as CD36, FABP1, ATGL, HSL, ACSL1, and ACSL5 in WD- and WD/CCl_4_-induced mice (Figures 3C, D). Therefore, the inhibition of *de novo* lipogenesis through downregulating the expressions of ACC and FAS might be at least one of the mechanisms for berberine to alleviate NAFLD, which is distinguished from that of bicyclol, and finally co-contribute to the enhancement of therapeutic effect against NAFLD when combined with berberine and bicyclol.

### Berberine But Not Bicyclol Modulates the Composition of Gut Microbiota

**FIGURE 5:**
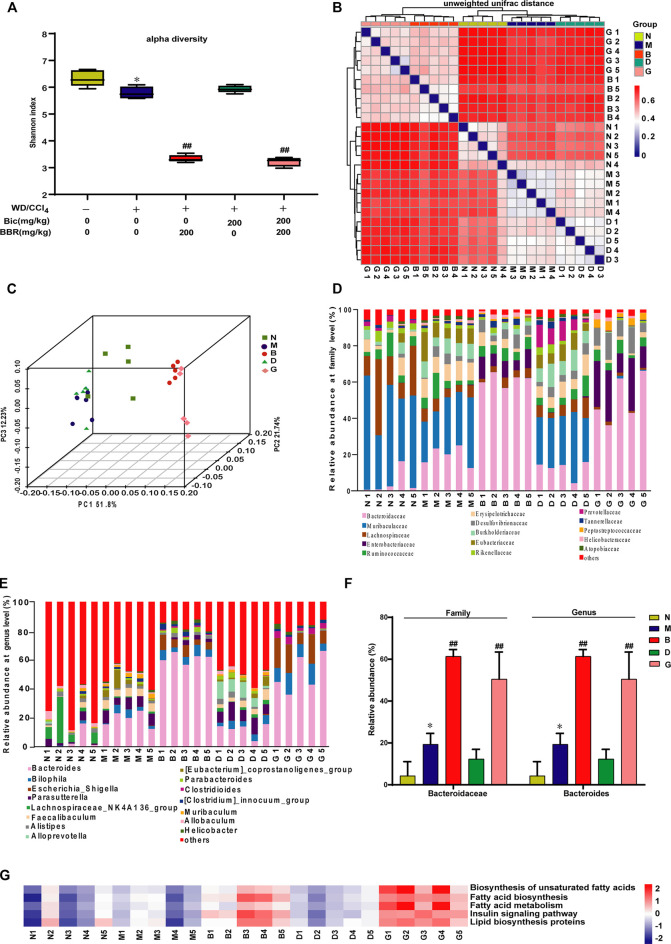
Berberine but not bicyclol modulates the composition of gut microbiota in WD/CCl_4_-induced NASH mice. A total of 25 male C57BL/6J mice fecal samples with five in each representative group were collected and analyzed using 16S rRNA gene sequencing. **(A)** The alpha diversity of each group was obtained using the Shannon index. **(B–C)** The beta diversity among samples was obtained using the unweighted UniFrac diversity distance **(B)** and PCoA **(C)** of gut microbiota. **(D–E)** The relative abundance of bacteria at family **(D)** and genus **(E)** level. **(F)** Representative histogram of the most abundant gut microbiota at the family and genus level. **(G)** Prediction of lipid metabolism-related microbiome function based on KEGG database. *p < 0.05, **p < 0.01 vs. the control group; ^#^p < 0.05, ^##^p < 0.01 vs. the WD/CCl_4_ group. N, Control; M, WD/CCl_4_; B, WD/CCl_4_+BBR(200 mg/kg); D, WD/CCl_4_+Bic(200 mg/kg); G, WD/CCl_4_+BBR(200 mg/kg)+Bic(200 mg/kg).

Because berberine might also possess physiological functions *via* regulation of gut microbiota, to further analyze the different roles of berberine and bicyclol in the treatment of NAFLD, the NASH mice induced by WD/CCl_4_ at the end of the experiment were carried out to analyze the diversity and evenness of gut microbiota. The results showed that the Shannon index was significantly lower in the WD/CCl_4_ group than in the control group; berberine but not bicyclol treatment further changed the index and bicyclol also did not affect the role of berberine, as presented in the combined treatment group (Figure 5A). Next, compared with the model group, the unweighted UniFrac distances (Figure 5B) and PCoA plot (Figure 5C) showed a shift in the overall gut microbiota in the berberine or combination group, while the bicyclol monotherapy group showed no obvious changes, suggesting their combined alleviation for NAFLD might partially derive from the gut microbiota regulation role of berberine.

Then, the composition of microbial community was further analyzed. Results showed that WD/CCl_4_ treatment led to moderate changes of community structure at the levels of bacteria family (Figure 5D) and genus (Figure 5E). Compared with the model group, the gut microbiota changed significantly in the berberine and combination groups, while bicyclol almost did not affect the structure (Figures 5D, E). Bacteroidaceae (family) and *Bacteroides* (genus) were reported to be associated with beneficial effects for improving biochemical parameters and hepatic fat fraction (Lang et al., 2020; Jobira et al., 2021). In our study, among the top 15 changed gut microbiota by the treatment of berberine, the ratio of Bacteroidaceae (family) and *Bacteroides* (genus) accounts for the most abundant, and they were significantly increased after berberine but not bicyclol treatment (Figure 5F). The correlation of microbial community with lipid metabolism was also predicted through the KEGG databases, and berberine but not bicyclol significantly changed the abundance of lipid metabolism-related microbial community (Figure 5G). Overall, these results suggested that berberine might also alleviate NASH partially through regulating the lipid metabolisms *via* gut microbiota, while bicyclol almost had no effect on them and also did not disturb the effect of berberine during their combination in the NASH model.

### Combined Use of Bicyclol and Berberine Decreases Lipid Accumulation *via* Regulating Lipid Metabolism-Related Gene Expression in FFA-Induced HepG2 Cells

**FIGURE 6:**
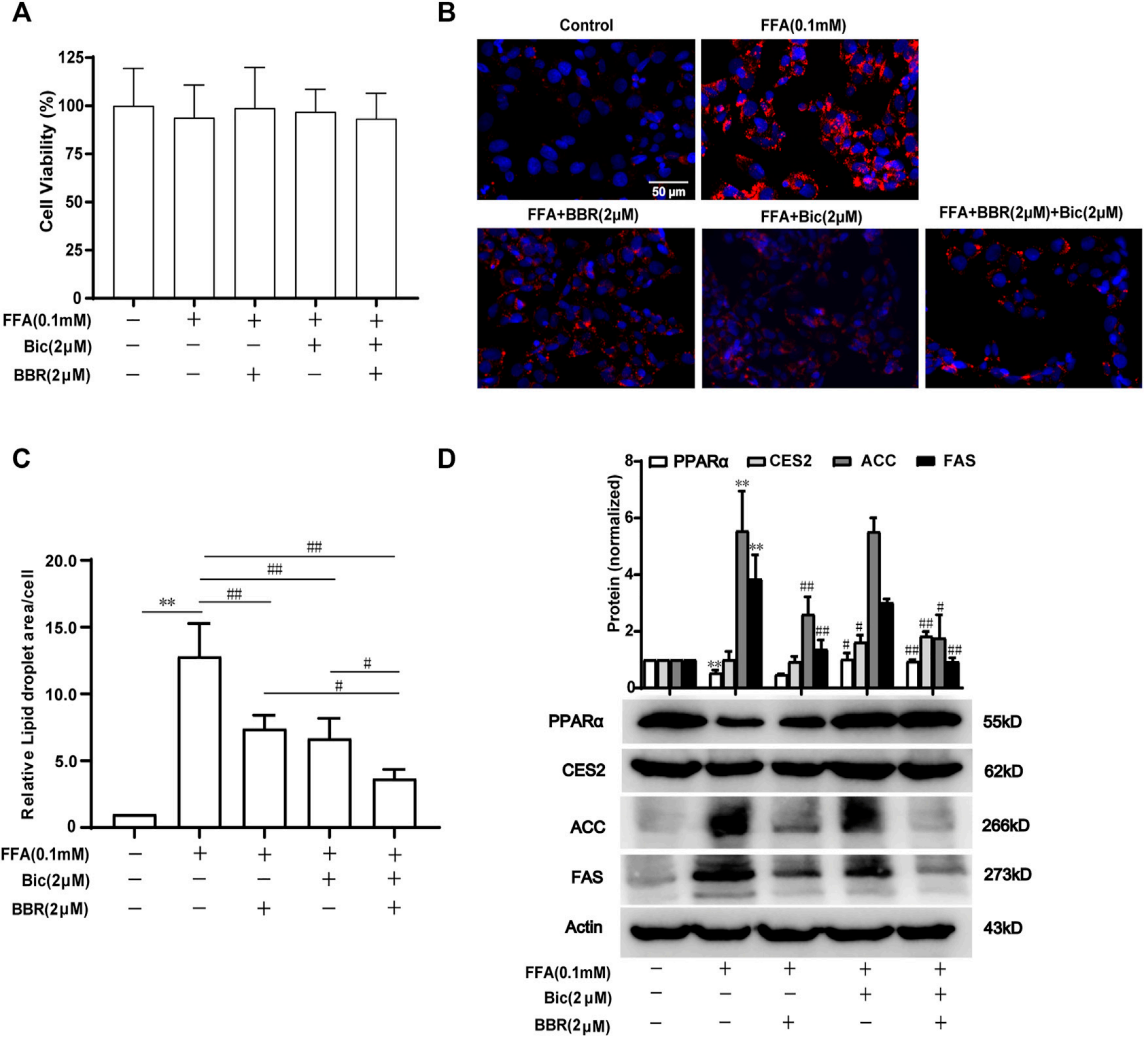
Combined use of bicyclol and berberine decreases lipid accumulation *via* regulating lipid metabolism-related gene expression in FFA-induced HepG2 cells. HepG2 cells were induced by 0.1 mM FFA and simultaneously treated for 24 h with 2 μM berberine, 2 μM bicyclol, or their combinations. The cells were assayed with an MTT method to show the cytotoxicity **(A)**, or carried out Nile Red staining to show LDs **(B)** and the level of LDs in cells were quantified using Image-Pro Plus 6.0 **(C)**. Total proteins were extracted and detected with Western blot **(D)**. *n* ≥ 3, **p < 0.01, the model group vs. control group; ^#^p < 0.05 and ^##^p < 0.01 vs. the model group or the monotherapy group. FFA, free fatty acid; LDs, lipid droplets; PPARα, proliferator-activated receptor α; CES2, carboxylesterase 2; ACC, acetyl-CoA carboxylase; FAS, fatty acid synthase.

To further verify the superior lipid-lowering effects and the mechanisms of action after combination of bicyclol and berberine, we established an FFA-induced lipid accumulation model in HepG2 cells. The MTT assay showed that 0.1 mM FFA and 2 μM bicyclol and/or berberine treatment had no obvious cytotoxicity on HepG2 cells (Figure 6A). Highly accumulated lipid droplets (LDs) in hepatocytes were observed by Nile Red staining after FFA induction, while the contents of LDs were dramatically reversed by berberine or bicyclol treatment, with more significant effects than their respective monotherapy after combination (Figure 6B). The effects were validated by the quantification of LDs (Figure 6C). The protein levels of PPARα and CES2 were slightly decreased by FFA induced-treatment but reversed by bicyclol with or without berberine (Figure 6D). In contrast, the protein expressions of ACC and FAS induced by FFA were decreased by berberine with or without bicyclol (Figure 6D). The results are consistent with the findings in mice models (Figures 3, 4), suggesting that the combined use of bicyclol and berberine preferably alleviates lipid accumulation *via* upregulating PPARα and CES2 expressions by bicyclol and downregulating ACC and FAS expressions by berberine.

## Discussion

**FIGURE 7:**
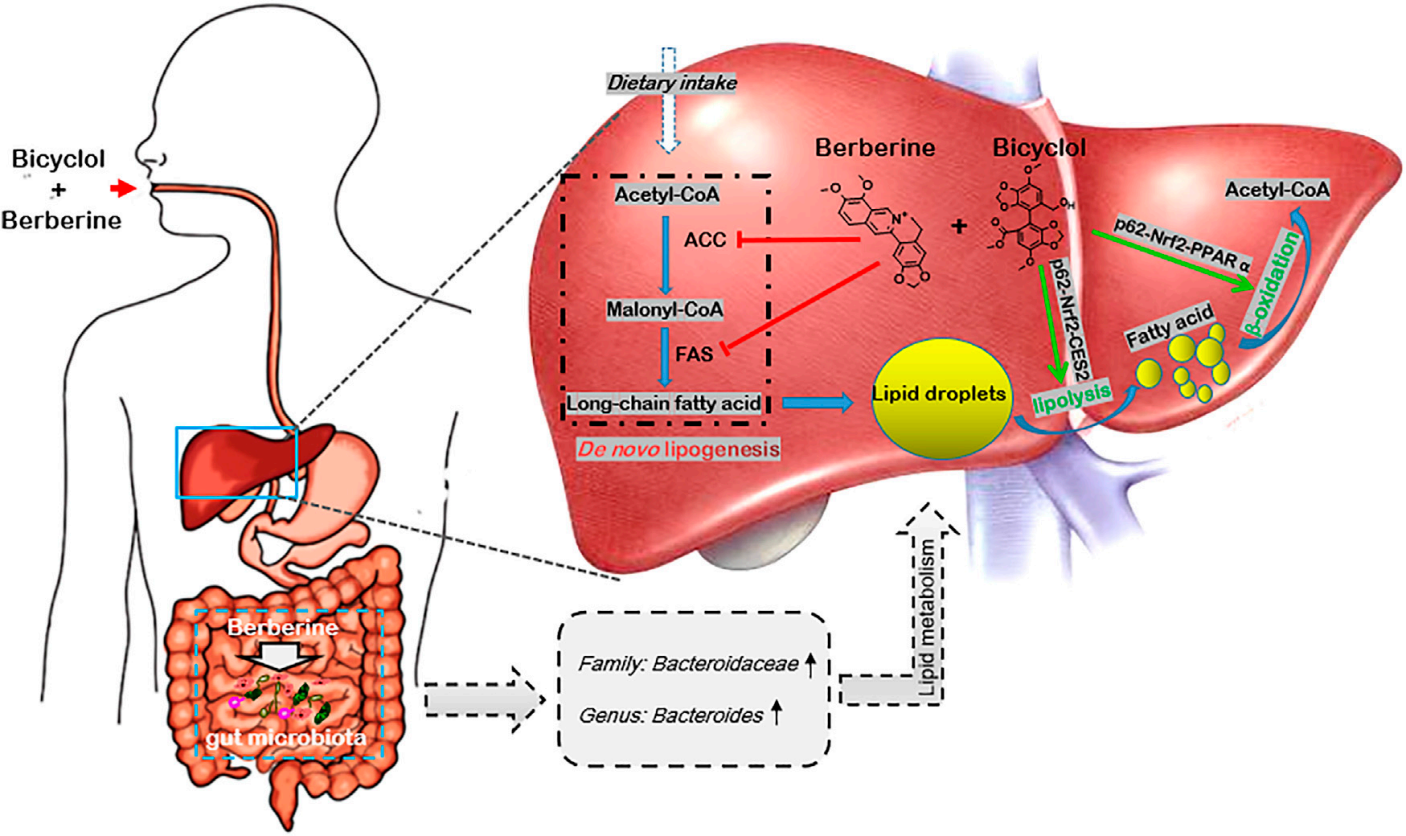
Schematic diagram of the therapeutic mechanism of combined use of bicyclol and berberine against NAFLD. Bicyclol enhanced lipolysis through p62-Nrf2-CES2 signaling axis and improved β-oxidation through p62-Nrf2-PPARα signaling axis, while berberine suppressed the *de novo* lipogenesis by inhibiting protein expressions of ACC and FAS, along with the regulation of lipid metabolisms *via* the gut microbiota, and thus co-facilitating to ameliorate the occurrence and progress of NAFLD. The green arrow shows a stimulatory effect, the red flathead shows an inhibitory effect.

NAFLD is a multisystem disease with comprehensive causative mechanisms and therefore refers to MAFLD (Eslam et al., 2020; Liu et al., 2020). Candidate drugs underlying clinical trials with single-mechanism of action were generally unsatisfied (Brown et al., 2021). In this study, we demonstrated that combined use of bicyclol and berberine exerted better preventive effects in WD-induced mouse steatosis and also presented superior therapeutic results in WD/CCl_4_-induced mouse NASH. The combination of bicyclol and berberine also showed better lipid-lowering effects in FFA-induced HepG2 cells. Mechanism study showed that bicyclol enhanced the lipolysis and β-oxidation through the p62-Nrf2-CES2/PPARα axis, while berberine suppressed the lipogenesis-related protein expressions of ACC and FAS, along with regulation of lipid metabolisms *via* gut microbiota (Figure 7). Importantly, co-treatment with them did not influence the pharmacologic roles of each other but enhanced the overall efficacy on NAFLD. The results validated our putative therapeutic strategy of combined use of bicyclol and berberine to treat NAFLD in the future as they have been approved, respectively, to use in clinic.

The early stage of NAFLD is mainly characterized by excessive lipid deposition in the liver, while the advanced stage of NAFLD is generally accompanied by fibrosis. In this study, we applied the Western diet (high in fat, sugar, and cholesterol) to mimic the diets in the real world, which might lead to a higher incidence of NAFLD (Lytle and Jump, 2016; Tsuchida et al., 2018). To assess the safety and preventive effect of the combined use of bicyclol and berberine, we first used a mild to moderate NAFLD mouse model induced by 16 weeks of intake of WD. Results showed that the combined use of the two drugs is rather safe, which is consistent with previous respective preclinical and clinical studies (Liu, 2009; Lan et al., 2015; Li et al., 2020). Of note, mouse body weight was slightly decreased after drug treatment compared with the WD-induced model group, though food intake was no difference. The slightly decreased body weight also predicted their pharmacological effects, which were present in the reduced indicators of lipid deposition (Figure 1). The combined effect in the WD/CCl_4_-induced NASH mouse model is similar to that in the preventive experiment (Figure 2), suggesting the potential application in the preventive and therapeutic treatment of NAFLD. Overall fibrosis state most likely progresses to hepatocellular carcinoma (HCC), and the combined use of bicyclol and berberine showed efficacy on the decrease of the NAS score after induction by WD/CCl_4_ for 12 weeks (Figure 2). Furthermore, bicyclol and berberine are, respectively, effective on HCC caused by various etiological factors (Sun et al., 2012; Xu et al., 2019). Therefore, the combined use might also be beneficial for the treatment of NAFLD-related HCC.

We previously carried out a meta-analysis study and demonstrated that bicyclol monotherapy could improve liver function and dyslipidemia in patients with NAFLD (Li et al., 2020), though the conclusion needs to be further verified with more well-designed and implemented studies, and the related mechanism study is limited. Yu et al. found that bicyclol protected against tetracycline-induced fatty liver mainly through ameliorating mitochondrial function and modulating the disturbance of PPARα-related genes (Yu et al., 2009). In contrast, Yao et al. reported that the anti-apoptosis associated with ER stress in this model might be the mechanism for bicyclol to attenuate fatty liver (Yao et al., 2016). Recently, bicyclol was reported to alleviate HFD-induced NAFLD through its anti-inflammatory mechanism (Zhao et al., 2021). Our results showed that the expression of lipolysis-related protein CES2 was decreased in WD or WD/CCl_4_-induced mice, and bicyclol reversed CES2 expression to improve NAFLD, which is consistent with the fact that CES2 is reduced in high fat diet-induced obese mice, diabetic db/db mice, and NASH patients, while the liver disease was alleviated after CES2 was restored (Li et al., 2016; Xu et al., 2021). Therefore, bicyclol might be contributory to its protective roles in NAFLD *via* restoring CES2. Meanwhile, bicyclol also enhanced the PPARα-mediated β-oxidation, and this role was also verified by previous report (Yu et al., 2009). Generally, CES2 was independent of the expression of PPARα (Wen et al., 2019), while all of them could be regulated by the pleiotropic transcription factor Nrf2 (Zhang et al., 2012; Baker et al., 2015; Tao et al., 2021). Nrf2 induced the downstream PPARα expression and was regulated by its upstream increased p62 to connect with Keap1 (Zhang et al., 2012; Baker et al., 2015; Lee et al., 2020; Tao et al., 2021). In our study, p62 and Nrf2 were decreased in WD- and WD/CCl_4_ treated mice and reversed by bicyclol (Figure 3). Altogether, the preventive and therapeutic role of bicyclol in NAFLD is related with the restoration of p62-Nrf2-CES2/PPARα signaling axis.

In contrast, the effects and mechanisms of berberine on NAFLD were well documented in various models, which were related to the increase of insulin sensitivity, regulation of the AMPK pathway, improvement of mitochondrial function, alleviation of oxidative stress, stabilization of LDLR mRNA, and regulation of gut microenvironment (Zhu et al., 2016). In this study, we verified the marked inhibition for critical fatty acid biosynthesis-related enzymes ACC and FAS in the NAFLD mouse models (Figure 4), which was consistent with the results in HepG2 cells directly treated with berberine (Cao et al., 2013). Encouragingly, ACC and FAS inhibitors were processed into clinical trials for treatment of patients with NAFLD (Loomba et al., 2018; Syed-Abdul et al., 2020). Consistent with the effects in animal experiments, the lipid-lowering effects and regulation for the key lipid-metabolism associated gene expressions of PPARα, CES2 by bicyclol and ACC, and FAS by berberine were verified in FFA-treated HepG2 (Figure 6). On the other hand, berberine possessed antimicrobial activity in clinic, and evidence indicated that the regulation for the gut microenvironment by berberine partially accounts for the improved NAFLD condition. For example, berberine increased protective bacteria like *Bifidobacteria* and decreased Gram-negative bacteria like *Escherichia coli*, which resulted in decrease of LPS release, TLR4/TNF-α activation, and insulin resistance (Liu et al., 2018). Berberine also enriched the short-chain fatty acid-producing bacteria and reduction of microbial diversity, which contribute to the treatment for high-fat diet-induced obesity in rats (Zhang et al., 2015). In this study, we also observed significant changes of gut microbiota after treatment by berberine but not bicyclol (Figure 5), and the most abundant microbiota are the lipid metabolism-related Bacteroidaceae (family) and *Bacteroides* (genus) in the berberine-treated groups (Figure 5F). We speculated that the increase of Bacteroidaceae (family) and *Bacteroides* (genus) might account for the beneficial role of berberine for NAFLD, because they were positively associated with a higher serum high-density lipoprotein (HDL) and lower levels of ALT, gamma-glutamyl-transferase (GGT), and ferritin (Lang et al., 2020) and negatively correlated with high hepatic fat fraction in patients with NAFLD (Jobira et al., 2021).

In summary, we first proposed a new available strategy of combined use of bicyclol and berberine to treat NAFLD. The better overall effect on NAFLD is related to the enhancement of lipolysis and β-oxidation by bicyclol *via* restoring the p62-Nrf2-CES2/PPARα signaling axis and suppression of lipogenesis by berberine *via* downregulating ACC and FAS, along with the enrichment of lipid metabolism-related Bacteroidaceae (family) *and Bacteroides* (genus) by berberine. Notably, the combined use of bicyclol and berberine does not influence the pharmacological roles of each other but enhances the amelioration effect for NAFLD, which predicts it to be a new available strategy to treat NAFLD.

## Data Availability

The original contributions presented in the study are publicly available. This data can be found here: PRJNA795575.
