# Supplementary figures and images for: Combined Use of Bicyclol and Berberine Alleviates Mouse Nonalcoholic Fatty Liver Disease
Source: Front Pharmacol. 2022 Feb 16;13:843872. doi: 10.3389/fphar.2022.843872 (PMC8889073; doi:10.3389/fphar.2022.843872)

**Figure 3A**

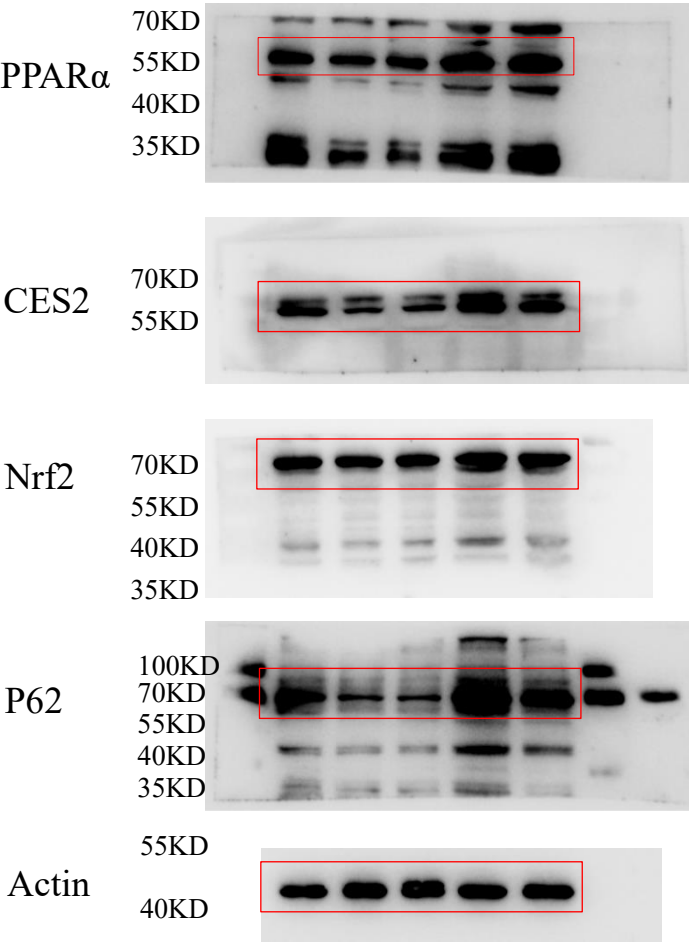

**Figure 3B**

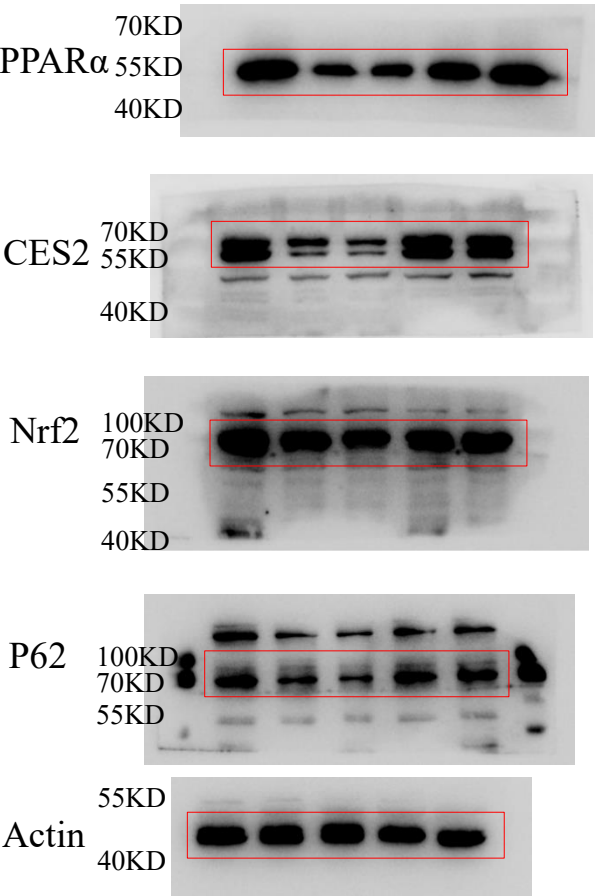

Figure 3C

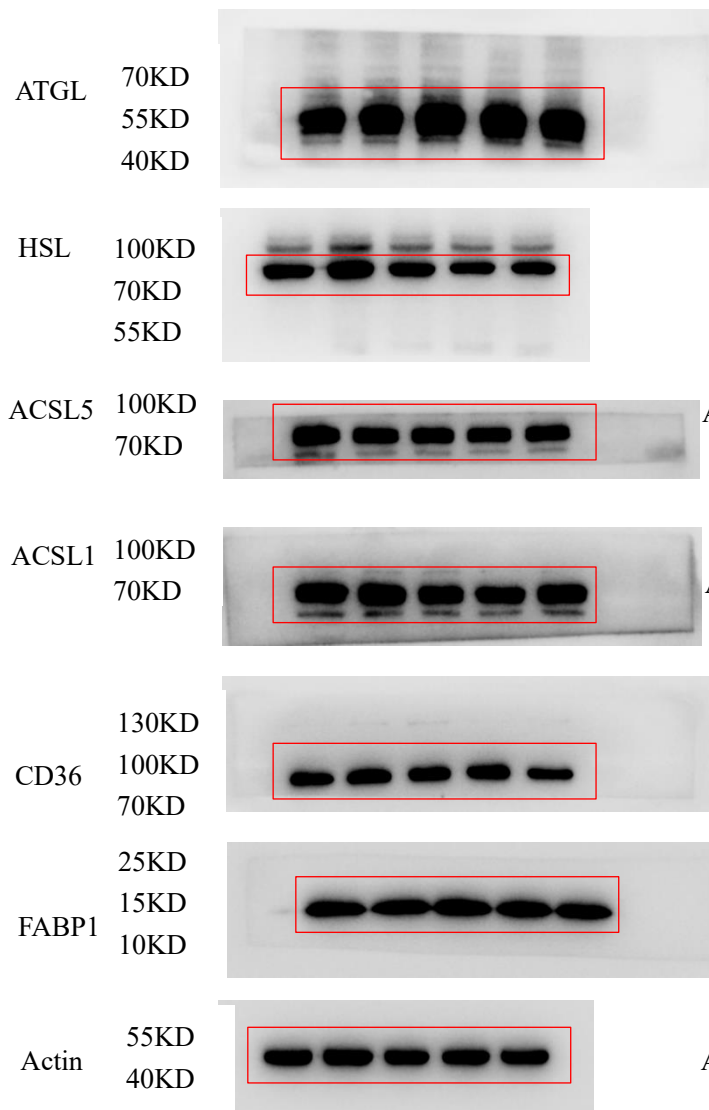

Figure 3D

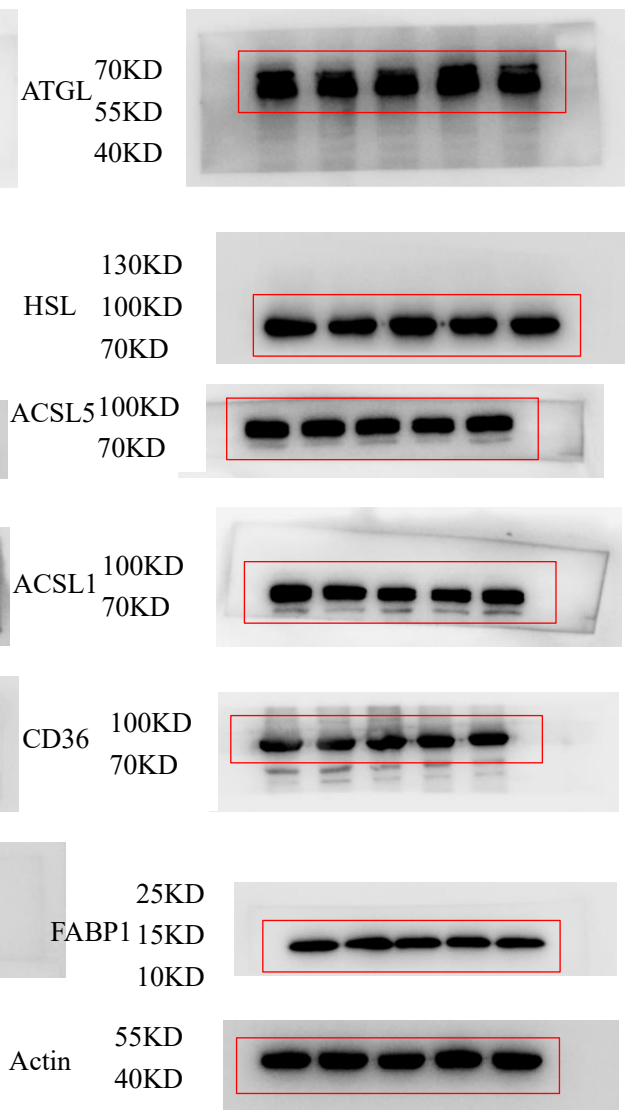

Figure 4A

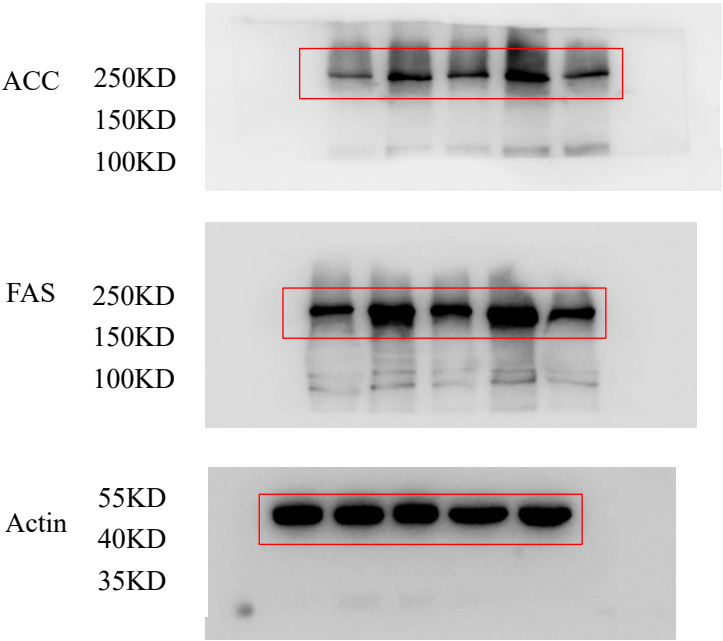

Figure 4B

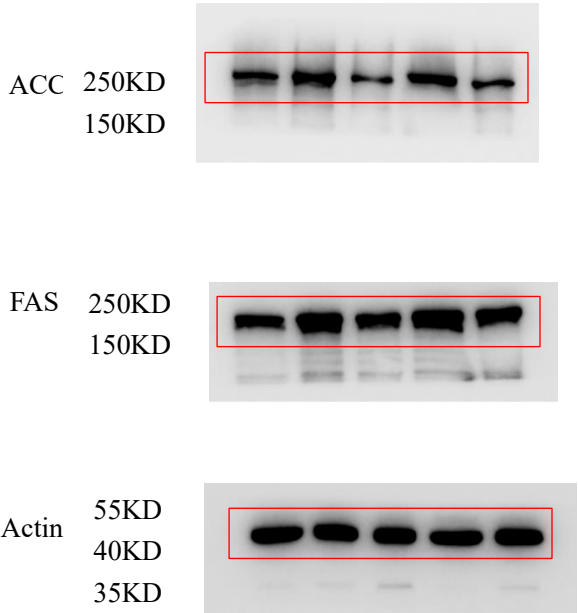

Figure 6D

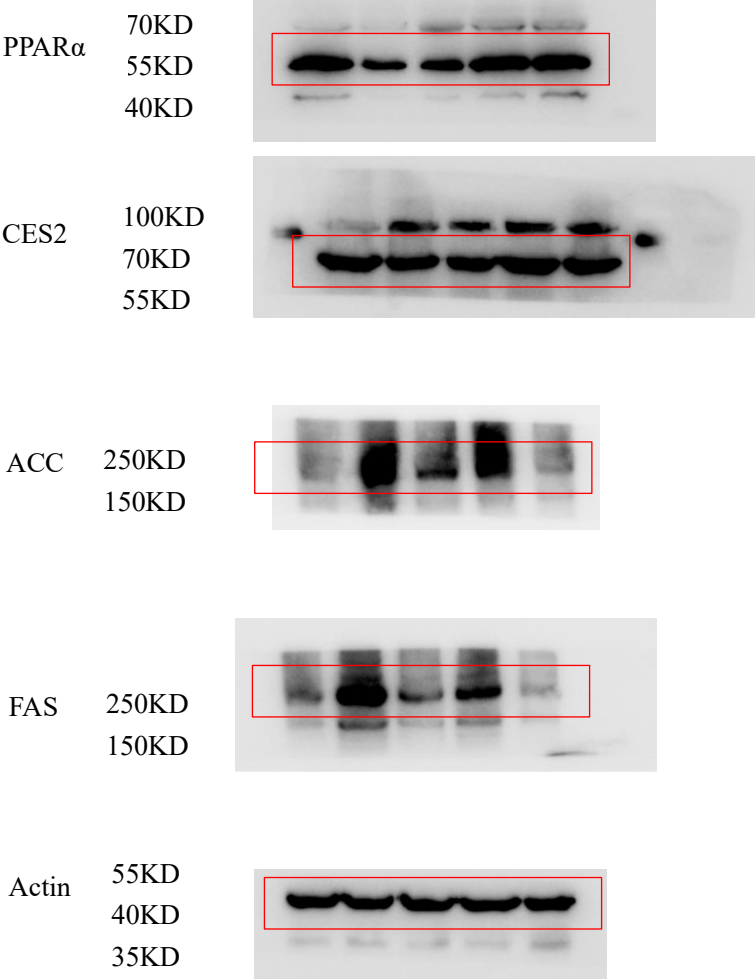

Supplement: Supplementary file 1 [file DataSheet1.PDF]
